# Heart failure awareness and influencing factors among adults in the Jazan, Saudi Arabia

**DOI:** 10.1097/MD.0000000000048822

**Published:** 2026-05-15

**Authors:** Luai Alhazmi, Omar Oraibi, Tariq Al Bahhawi, Ali I. Alharbi, Ali A. Zalah, Faisal H. Almalki, Sawsan H. Alsharif, Alhassan H. Hobani, Abdulrahman Hakami, Mohammed Ali Madkhali, Mohammed Somaili, Ahmed Sayed, Mohammed A. Jeraiby, Amal H. Mohamed, Mostafa Mohrag, Mohamed Salih Mahfouz

**Affiliations:** a Department of Internal Medicine, College of Medicine, Jazan University, Jazan, Saudi Arabia; b Department of Family and Community Medicine, College of Medicine, Jazan University, Jazan, Saudi Arabia; c Internal Medicine Department, King Fahad Central Hospital, Jazan, Saudi Arabia; d Radiology Department, King Fahad Medical City, Riyadh, Saudi Arabia; e Department of Orthopedic Surgery, King Fahad Central Hospital, Jazan, Saudi Arabia; f Department of Basic Medical Science, College of medicine, Jazan University, Saudi Arabia.

**Keywords:** awareness, heart failure, influencing factors, knowledge, risk factors

## Abstract

Heart failure (HF) is a condition in which the heart cannot pump enough blood to meet the body’s requirements, causing multiple significant symptoms that affect the patient’s quality of life. Worldwide, HF awareness is known to be low. However, no studies have assessed the levels of awareness of HF and their influencing factors in our region. This study aims to investigate the knowledge of HF and its influencing factors among adults in Jazan, Saudi Arabia. A community-based descriptive cross-sectional study was conducted in Jazan, Saudi Arabia. Adult participants (≥18 years) were recruited using an online, self-administered structured questionnaire. The questionnaire assessed sociodemographic characteristics, knowledge of HF definition, symptoms, causes, risk factors, and treatment options. Descriptive statistics were used to summarize participants’ responses, and logistic regression analysis was performed to identify factors associated with higher HF knowledge. Out of 399 respondents, more than half 51.6% identified HF as a condition involving the heart’s pumping failure. As far as symptoms are concerned, only 16.3% recognized shortness of breath, about 1.8% noted reduced exercise tolerance, and only 1.5% identified leg edema as typical symptoms of HF. About 45.6% of the participants were aware of all 3 symptoms. Moreover, participants with higher education exhibited higher levels of knowledge. Less than half of the participants correctly identified cardiovascular conditions leading to HF 45.9%. Knowledge regarding HF treatment was also insufficient; some participants believed that HF has no effective treatment or requires prolonged bed rest. Additionally, working in the medical field was significantly associated with better knowledge about HF (odds ratio = 3.38, 95% confidence interval: 2.11–5.54). The study found inadequate knowledge among participants, particularly regarding the most common causes of HF and the measures to reduce the risk of developing it. These findings highlight the need for improved education and awareness programs to address misconceptions and provide accurate information about HF and its management.

## 1. Introduction

Heart failure (HF) is described by the American Heart Association (AHA) as “a complex clinical syndrome that results from any structural or functional impairment of ventricular filling or blood ejection.”^[1]^ HF is recognized as a significant global health concern. For instance, data from the Global Burden of Diseases 2019 reported global age-standardized rates for HF as 711.90 per 1,00,000 population (95% uncertainty interval [UI]: 591.15–858.29) for prevalence and 63.92 per 1,00,000 population (95% UI: 41.49–91.95) for years lived with disability (YLD). HF also imposes a substantial economic burden, with an estimated annual cost of $108 billion.^[2]^

Saudi Arabia was among the countries with the highest percentage increase in age-standardized point prevalence, recording a rise of 20.97% between 1990 and 2019, as reported by the Global Burden of Disease study.^[3]^ Furthermore, it ranked as one of the highest-spending nations on HF, with an annual expenditure of $648 million, accounting for 0.60% of the total global HF spending.^[2]^ Local research indicated that the age range of individuals diagnosed with HF ranged from 55 to 61 years.^[4]^ This age range is approximately 10 years younger than the average observed in industrialized nations such as the United States and Western European countries.^[5-8]^

The prevalence of cardiovascular disease (CVD) in Saudi Arabia is anticipated to be considerably elevated due to the increasing incidence of CVD risk factors such as diabetes mellitus, obesity, hypertension, dyslipidemia, and others.^[9]^ These variables have increased in Saudi Arabia, intensified by a sedentary lifestyle. Recent generations have predominately embraced a sedentary lifestyle. This phenomenon is likely multifaced, attributable to environmental weather conditions restricting outdoor activities, coupled with the effects of globalization, including the prevalence of social media, video game usage, and a cosmopolitan diet high in fats and sugars. The progressively sedentary lifestyle of Saudis adversely affected their well-being. Recent studies estimate that HF affects approximately 1% to 2% of the population in Saudi Arabia, with around 4,55,222 individuals living with the condition. Additionally, acute coronary syndrome remains a significant contributor to the burden of HF, highlighting the critical role of biomarkers in improving management and patient outcomes in the region.^[10-12]^

Despite significant advances in HF management in recent years, knowledge and awareness of HF remain relatively understudied. Raising awareness about HF is crucial, as improved awareness may facilitate earlier recognition of symptoms, timely healthcare-seeking behavior, and better adherence to preventive strategies and treatment plans, which collectively may help reduce disease progression, readmission rates, and premature mortality associated with HF. For instance, the Study of Heart failure Awareness and Perception in Europe (SHAPE) study conducted in 2005 among the general public in 9 European countries revealed very low awareness levels; only 3% of participants could identify HF symptoms, compared to 31% for angina and 51% for ischemic stroke.^[13]^ In Saudi Arabia, a study conducted in the Makkah region found that only 24.1% of participants correctly recognized HF symptoms.^[14]^ Another study revealed that <50% of respondents had even heard of HF.^[15]^

Given the increasing burden of HF in Saudi Arabia, identifying knowledge gaps in different regions is critical for developing targeted public health interventions. Understanding population-level awareness can inform the design of educational initiatives and preventive strategies that are better aligned with local needs and misconceptions, particularly in settings where cultural, lifestyle, and healthcare access factors vary. While numerous international studies and meta-analyses on HF exist, research on public awareness and perceptions remains scarce, particularly in Middle Eastern populations. The need for region-specific data is further justified by variations in healthcare access, lifestyle factors, and cultural beliefs about CVD. This study sought to answer the following research question: What is the level of awareness and knowledge regarding HF among adults in the Jazan region of Saudi Arabia, and which sociodemographic factors are associated with appropriate HF knowledge? The objectives of this study were to assess the level of knowledge and awareness of HF among adults in the Jazan region, to evaluate awareness of HF symptoms, causes, risk factors, and treatment options, and to identify sociodemographic and clinical factors associated with appropriate HF knowledge. Understanding public awareness levels in this region will provide valuable insights for designing effective health education programs and policies tailored to the local population.

## 2. Materials and methods

### 2.1. Study design and subject selection

This cross-sectional study utilized an online questionnaire and was conducted between August 2023 and November 2023. Inclusion criteria were adults aged 18 years or older residing in the Jazan region, Saudi Arabia, who agreed to participate and completed the questionnaire. Exclusion criteria included individuals younger than 18 years, those who declined participation, submitted incomplete questionnaires, or were unable to provide informed consent. This observational study was conducted in accordance with the Strengthening the Reporting of Observational Studies in Epidemiology (STROBE) guidelines.

### 2.2. Sample size and data collection

To determine the appropriate sample size, we utilized the Cochrane formula, expressed as n = *Z*^2^*p* (1 − *p*)/*d*^2^. In this formula, n stands for the sample size, *Z* represents the critical value for a 95% confidence interval, *p* is the estimated proportion (set at 50%), and *d* denotes the margin of error (set at 5%). Initially, the calculation indicated a minimum sample size of 384. However, to enhance the reliability of the results, we opted to increase the sample size to 399 respondents. The study questionnaire was distributed to approximately 492 eligible participants, of whom 399 individuals completed the survey, resulting in a response rate of 81%. The questionnaire used in this study was adapted from previously validated tools used in similar research on HF awareness.^[16,17]^ Minor modifications were made to ensure cultural relevance, clarity of wording, and suitability for the local population in Jazan, based on expert input from local healthcare professionals. The final version underwent a pilot test with 30, who were not included in the final analysis, to assess clarity and reliability. We sent the electronic survey across various social media platforms, such as WhatsApp, X and Telegram. The questionnaire included 22 items to evaluate the study’s objectives and is provided as Supplementary Material, Supplemental Digital Content. The initial section of the questionnaire focused on collecting demographic information from participants, encompassing 6 items: age, sex, residency, educational level, occupation, and whether they belonged to a medical profession. A medical profession was defined as any occupation involving formal healthcare training, including physicians, nurses, pharmacists, laboratory technicians, and other allied health professionals.

Two questions were included to assess participants’ exposure to HF. To evaluate understanding of HF, 6 questions addressed symptoms and causes, while 2 questions measured awareness of HF prevalence. Additionally, 3 questions were designed to assess knowledge of HF care and prevention, with the final 3 questions addressing angina pectoris, stroke, and HF collectively. Knowledge levels score were categorized as either “inappropriate < 60%” or “appropriate ≥ 60,” and the corresponding percentages were reported for each category

### 2.3. Statistical analysis

All analyses were performed using R software (version 4.4.1; R Foundation for Statistical Computing, Vienna, Austria). Descriptive statistics were used to summarize participants’ characteristics, and results were reported as frequencies and percentages. Differences in HF awareness between males and females, as well as associations between sociodemographic factors and knowledge level, were examined using the chi-square test or Fisher exact test when appropriate, with statistical significance set at *P* < .05.

For inferential analysis, binary logistic regression was conducted in 2 stages. Initially, bivariate (unadjusted) models were employed to investigate the raw associations between each independent variable and knowledge level. Second, all relevant variables were entered into a multiple logistic regression model to identify independent predictors of appropriate knowledge. The dependent variable was HF knowledge level (appropriate vs inappropriate). Covariates were prespecified and classified as sociodemographic (age, sex, nationality, education level, income, and residence), occupational (employment status and work in the medical field), and clinical factors (personal and family history of HF). All variables were analyzed as categorical using clinically or sociodemographically meaningful groupings, with detailed category definitions provided in the tables. Covariates were selected a priori based on clinical relevance and prior literature as potential confounders. Odds ratios (ORs), adjusted odds ratios (AORs), and their corresponding 95% confidence intervals (CIs) were reported

Prior to analysis, data were screened for completeness and consistency. Questionnaires with substantial missing responses were excluded from the final dataset. Categorical variables were coded numerically for statistical analysis. Knowledge scores were calculated based on the proportion of correct responses and categorized as appropriate (≥60%) or inappropriate (<60%), consistent with previous studies. No data imputation techniques were applied. Comparative analyses were conducted to examine differences in HF awareness across key subgroups. Associations between sociodemographic variables and knowledge level were first assessed using univariable analyses, followed by multivariable logistic regression to identify independent predictors while controlling for potential confounders.

### 2.4. Ethical consideration

The study received approval from the Scientific Research Ethics Committee (REC) at Jazan University under the reference number REC-44/07/541. Informed consent was obtained from all participants in accordance with Saudi Arabian ethical guidelines before commencing the anonymous questionnaire survey. Participants had the right to withdraw from the survey at any point during the research process. To maintain privacy and confidentiality, no identifying information, including email addresses or IP addresses, was collected.

## 3. Results

### 3.1. Sociodemographic characteristics

Out of the 492 individuals invited to participate in the survey, a total of 399 respondents completed the questionnaire, yielding a response rate of 81%. Table 1 summarizes the demographic characteristics of the 399 participants. Most respondents were between 20 and 29 years of age (38.6%), and over half were female (52.6%). The majority were Saudi nationals (96.2%), with nearly equal proportions of married (47.6%) and single participants (47.9%). Most resided in cities or villages (85.7%), and a large proportion had a university-level education (68.9%). Employees represented 46.6% of the total, followed by students (35.1%). Students who were also employed were classified under “Employee” if working >20 h/wk; otherwise, they were classified as “Student.” Approximately one-third reported a monthly income above 9000 Saudi Riyals (SR; 33.1%) and 44.6% <3000 SR. While 28.3% worked in the medical field, 58.6% had a family member working in healthcare. Only 1.3% reported having HF themselves, and 15.8% had a family member with HF.

**Table 1:** Demographic of study participants (n = 399).

Variables		Overall n = 399 n (%)	Male n = 189	Female n = 2010
Age (yr)	<20	46 (11.5)	19 (10.1%)	27 (12.9%)
	20–29	154 (38.6)	82 (43.4%)	72 (34.3%)
	30–39	78 (19.5)	39 (20.6%)	39 (18.6%)
	40–49	93 (23.3)	32 (16.9%)	61 (29.0%)
	50–59	28 (7.0)	17 (9.0%)	11 (5.2%)
Nationality	Saudi	384 (96.2)	179 (94.7%)	205 (97.6%)
	Non-Saudi	15 (3.8)	10 (5.3%)	5 (2.4%)
Social status	Married	190 (47.6%)	90 (47.6%)	100 (47.6%)
	Single	191 (47.9%)	96 (50.8%)	95 (45.2%)
	Widow/divorced	18 (4.5%)	3 (1.6%)	15 (7.1%)
Residence	City	174 (43.6%)	89 (47.1%)	85 (40.5%)
	Village	168 (42.1%)	72 (38.1%)	96 (45.7%)
	Mountains	57 (14.3%)	28 (14.8%)	29 (13.8%)
Level of education	University	275 (68.9%)	136 (72.0%)	139 (66.2%)
	Diploma	13 (3.3%)	4 (2.1%)	9 (4.3%)
	High school	92 (23.1%)	40 (21.2%)	52 (24.8%)
	Below high school	19 (4.8%)	9 (4.8%)	10 (4.8%)
Occupation	Employee	186 (46.6%)	102 (54.0%)	84 (40.0%)
	Student	140 (35.1%)	59 (31.2%)	81 (38.6%)
	Nonworking	73 (18.3%)	28 (14.8%)	45 (21.4%)
Income	<3000 SR	178 (44.6%)	72 (38.1%)	106 (50.5%)
	3000–6000 SR	38 (9.5%)	21 (11.1%)	17 (8.1%)
	6000–9000 SR	51 (12.8%)	27 (14.3%)	24 (11.4%)
	>9000 SR	132 (33.1%)	69 (36.5%)	63 (30.0%)
Do you work in the medical field?	Yes	113 (28.3%)	67 (35.4%)	46 (21.9%)
	No	286 (71.7%)	122 (64.6%)	164 (78.1%)
Family member working in the medical field?	Yes	234 (58.6%)	113 (59.8%)	121 (57.6%)
	No	165 (41.4%)	76 (40.2%)	89 (42.4%)
Do you have heart failure?	Yes	5 (1.3%)	3 (1.6%)	2 (1.0%)
	No	394 (98.7%)	186 (98.4%)	208 (99.0%)
Do you have heart failure patient in the family?	Yes	63 (15.8%)	33 (17.5%)	30 (14.3%)
	No	336 (84.2%)	156 (82.5%)	180 (85.7%)

n = number, SR = Saudi Riyal.

### 3.2. Awareness about HF

Awareness of HF varied considerably across domains (Table 2). Less than half of the participants correctly identified cardiovascular conditions leading to HF (45.9%), and a similar proportion recognized major symptoms (45.6%). A substantial proportion selected “I don’t know” for disease causes (31.8%), symptom recognition (33.3%), or HF progression (52.4%). Sex differences were observed in several items based on univariable (unadjusted) chi-square analyses comparing males and females; no multivariable adjustment was applied for these item-level comparisons: females showed better recognition of disease causes (*P* = .02), the most common cause of HF (*P* = .008), chest heaviness attributable to angina (*P* = .020), and neurological signs indicative of stroke (*P* = .005). For most remaining domains, awareness levels were comparable between males and females.

**Table 2:** Awareness of heart failure among the study participants according to gender.

Questions		All participants (n = 399)	Gender		*P*-value*
			Male n = 189	Female n = 210	
		n (%)	n (%)	n (%)	
Which disease/diseases can lead to heart failure?	Underlying cardiovascular conditions	30 (7.5)	17 (9.0)	13 (6.2)	.02
	Heart diseases in general	26 (6.5)	8 (4.2)	18 (8.6)	
	Angina or MI	27 (6.8)	10 (5.3)	17 (8.1)	
	lung disorders	6 (1.5)	4 (2.1)	2 (1.0)	
	All mentioned diseases	183 (45.9)	77 (40.7)	106 (50.5)	
	I don’t know	127 (31.8)	73 (38.6)	54 (25.7)	
What are common symptoms of heart failure?	Shortness of breath under strain	65 (16.3)	31 (16.4)	34 (16.2)	.20
	Weakness of physical performance	7 (1.8)	5 (2.6)	2 (1.0)	
	Accumulation of water in the legs	6 (1.5)	4 (2.1)	2 (1.0)	
	Pain in right upper arm	6 (1.5)	5 (2.6)	1 (0.5)	
	All symptoms	182 (45.6)	78 (41.3)	104 (49.5)	
	I don’t know	133 (33.3)	66 (34.9)	67 (31.9)	
Which is the most common cause for heart failure?	Coronary blood artery disease with blood flow disruption	195 (48.9)	93 (49.2)	102 (48.6)	.008
	Lack of sleep	10 (2.5)	7 (3.7)	3 (1.4)	
	Genetic endowments	45 (11.3)	11 (5.8)	34 (16.2)	
	Weight loss	4 (1.0)	3 (1.6)	1 (0.5)	
	I don’t know	145 (36.3)	75 (39.7)	70 (33.3)	
How is HF caused?	Due to the heart’s inability to pump effectively	206 (51.6)	101 (53.4)	105 (50.0)	.40
	Blockage of a venous valve	12 (3.0)	3 (1.6)	9 (4.3)	
	Blockage of a brain vessel	16 (4.0)	5 (2.6)	11 (5.2)	
	By overweight	4 (1.0)	2 (1.1)	2 (1.0)	
	I don’t know	161 (40.4)	78 (41.3)	83 (39.5)	
What is the frequency of HF in the population?	There are only single cases	4 (1.0)	3 (1.6)	1 (0.5)	.10
	It is very rare (<0.1%)	29 (7.3)	7 (3.7)	22 (10.5)	
	A prevalent illness	120 (30.1)	58 (30.7)	62 (29.5)	
	Almost everyone will be affected	8 (2.0)	4 (2.1)	4 (1.9)	
	I don’t know	238 (59.6)	117 (61.9)	121 (57.6)	
Which age group is especially affected by HF?	Children of up to 12 yr	11 (2.8)	5 (2.6)	6 (2.9)	.30
	Adolescents of up to 18 yr	5 (1.3)	2 (1.1)	3 (1.4)	
	Adults of up to 50 yr	119 (29.8)	47 (24.9)	72 (34.3)	
	Adults above 65 yr	144 (36.1)	73 (38.6)	71 (33.8)	
	I don’t know	120 (30.1)	62 (32.8)	58 (27.6)	
How is the course of HF?	HF mostly goes away by itself	4 (1.0)	2 (1.1)	2 (1.0)	.30
	At least 1 mo of therapy heals it	39 (9.8)	22 (11.6)	17 (8.1)	
	Severe as malignant cancer	22 (5.5)	12 (6.3)	10 (4.8)	
	Surgery is required to heal it	125 (31.3)	50 (26.5)	75 (35.7)	
	I don’t know	209 (52.4)	103 (54.5)	106 (50.5)	
What can I do to reduce my risk of developing HF?	Healthy diet	13 (3.3)	6 (3.2)	7 (3.3)	.60
	Sufficient physical exercises	24 (6.0)	12 (6.3)	12 (5.7)	
	Not smoke	5 (1.3)	3 (1.6)	2 (1.0)	
	All the above measures	276 (69.2)	124 (65.6)	152 (72.4)	
	I don’t know	81 (20.3)	44 (23.3)	37 (17.6)	
What are the treatment options for HF?	There is no treatment	14 (3.5)	6 (3.2)	8 (3.8)	.80
	Absolute bed rest for months	32 (8.0)	13 (6.9)	19 (9.0)	
	Diet	5 (1.3)	2 (1.1)	3 (1.4)	
	Lifelong treatment with medicines	161 (40.4)	74 (39.2)	87 (41.4)	
	I don’t know	187 (46.9)	94 (49.7)	93 (44.3)	
What condition causes chest heaviness during exercise but not during rest?	Angina or MI	156 (39.1)	77 (40.7)	79 (37.6)	.020
	GIT disorders	6 (1.5)	0 (0.0)	6 (2.9)	
	Other heart diseases	87 (21.8)	32 (16.9)	55 (26.2)	
	lung disorders	37 (9.3)	20 (10.6)	17 (8.1)	
	I don’t know	113 (28.3)	60 (31.7)	53 (25.2)	
Facial paralysis, double vision, and acute arm unilateral weakness suggest what disease?	Stroke	117 (29.3)	67 (35.4)	50 (23.8)	.005
	Parkinson disease, epilepsy, other brain diseases	41 (10.3)	15 (7.9)	26 (12.4)	
	Other heart diseases	22 (5.5)	8 (4.2)	14 (6.7)	
	Angina or MI	46 (11.5)	13 (6.9)	33 (15.7)	
	I don’t know	173 (43.4)	86 (45.5)	87 (41.4)	
What disease do you think of if someone has breathlessness, tiredness, and swollen ankles?	Heart failure	182 (45.6)	80 (42.3)	102 (48.6)	.066
	Heart diseases in general	0 (0.0)	0 (0.0)	0 (0.0)	
	Angina or MI	52 (13.0)	29 (15.3)	23 (11.0)	
	lung disorders	46 (11.5)	16 (8.5)	30 (14.3)	
	I don’t know	119 (29.8)	64 (33.9)	55 (26.2)	

GIT = gastrointestinal tract, HF = heart failure, MI = myocardial infarction, n = number.

* *P*-value based on chi squared test/Fisher exact test; *P*-value < .05 that is considered statistically significant.

### 3.3. Knowledge level of HF and related influencing factors

Overall, 216 individuals (54.1%) demonstrated appropriate knowledge of HF (Table 3). Bivariate analyses indicated that appropriate knowledge was significantly associated with nationality, occupation, income, working in the medical field, and having a family member working in healthcare. Participants with higher income, those employed or studying, and individuals with medical-field exposure exhibited markedly higher levels of appropriate knowledge. No significant associations were found with age, sex, education, residence, or personal/family history of HF.

**Table 3:** Knowledge level of heart failure and related influencing factors among the general population in KSA’s southern regions (n = 399).

Variables		Knowledge level N (%)		*P*-value
		Inappropriate N = 183	Appropriate N = 216	
		N	N	
Age groups (yr)	<20	27 (15)	19 (8.8)	.30
	20–29	73 (40)	81 (38)	
	30–39	33 (18)	45 (21)	
	40–49	39 (21)	54 (25)	
	50 yr and above	11 (6.0)	17 (7.9)	
Gender	Male	91 (50)	98 (45)	.40
	Female	92 (50)	118 (55)	
Nationality	Saudi	181 (99)	203 (94)	.021
	Non-Saudi	2 (1.1)	13 (6.0)	
Social status	Married	82 (45)	108 (50)	.50
	Single	91 (50)	100 (46)	
	Widow/divorced	10 (5.5)	8 (3.7)	
Residence	City	77 (42)	97 (45)	.14
	Village	73 (40)	95 (44)	
	Mountains	33 (18)	24 (11)	
Level of education	University	115 (63)	160 (74)	.080
	Diploma	6 (3.3)	7 (3.2)	
	High school	50 (27)	42 (19)	
	Below high school	12 (6.6)	7 (3.2)	
Occupation	Employee	72 (39)	114 (53)	<.001
	Student	60 (33)	80 (37)	
	Not-working	51 (28)	22 (10)	
Income (SR)	<3000	95 (52)	83 (38)	<.001
	3000–6000	21 (11)	17 (7.9)	
	6000–9000	30 (16)	21 (9.7)	
	>9000	37 (20)	95 (44)	
Do you work in the medical field?	Yes	29 (16)	84 (39)	<.001
	No	154 (84)	132 (61)	
Family member working in the medical field?	Yes	92 (50)	142 (66)	.002
	No	91 (50)	74 (34)	
Do you have heart failure?	Yes	1 (0.5)	4 (1.9)	.50
	No	182 (99)	212 (98)	
Do you have HF patient in the family?	Yes	24 (13)	39 (18)	.20
	No	159 (87)	177 (82)	

HF = heart failure, KSA = Kingdome of Saudi Arabia, n = number, SR = Saudi Riyal.

* *P*-value based on chi squared test/Fisher exact test; *P*-value < .05 that is considered statistically significant.

### 3.4. Logistic regression analysis

Bivariate (unadjusted) logistic regression (Table 4) showed that being non-Saudi (OR = 5.80, 95% CI: 1.57–37.39), employed (OR = 3.67, 95% CI: 2.08–6.66), a student (OR = 3.09, 95% CI: 1.77–5.72), having a high income (>9000 SR; OR = 2.94, 95% CI: 1.83–4.79), and working in the medical field (OR = 3.38, 95% CI: 2.11–5.54) were significantly associated with appropriate knowledge. Age, sex, education, and HF exposure were not significant predictors in the unadjusted model.

**Table 4:** Binary logistic regression for the factors associated with study participants’ appropriate knowledge about heart failure (n = 399).

Factors		*B*	SE	Wald	*P* value	AOR	95% CI for AOR	
							Lower	Upper
Age	<20 (ref)							
	20–29	0.46	0.34	1.79	.181	1.58	0.81	3.11
	30–39	0.66	0.38	3.08	.079	1.94	0.93	4.1
	40–49	0.68	0.37	3.42	.064	1.97	0.97	4.07
	50 yr and above	0.79	0.49	2.59	.108	2.20	0.85	5.86
Sex (female)		0.17	0.20	0.75	.385	1.19	0.80	1.77
Nationality (non-Saudi)		1.76	0.77	5.26	.022	5.80	1.57	37.39
Occupation	Not-working (ref)							
	Employee	1.30	0.30	19.27	<.001	3.67	2.08	6.66
	Student	1.13	0.31	13.51	<.001	3.09	1.77	5.72
Income (SR)	<3000 (ref)							
	3000–6000	−0.08	0.36	0.05	.832	0.93	0.45	1.87
	6000–9000	−0.22	0.32	0.47	.491	0.80	0.42	1.50
	>9000 SR	1.08	0.25	19.33	<.001	2.94	1.83	4.79
Do you work in the medical field? (yes)		1.22	0.25	24.53	<.001	3.38	2.11	5.54
Education	Below high school (ref)							
	University	0.87	0.49	3.13	.077	2.39	0.93	6.58
	Diploma	0.69	0.73	0.90	.344	2.00	0.48	8.76
	High school	0.36	0.52	0.49	.483	1.44	0.53	4.18
Do you have HF? (yes)		1.23	1.12	1.21	.272	3.43	0.50	67.50
Do you have HF patient in the family? (yes)		0.38	0.28	1.81	.179	1.46	0.85	2.56

AOR = adjusted odds ratio, *B* = regression coefficient, CI = confidence interval, HF = heart failure, n = number, SE = standard error, SR = Saudia Riyals.

* *P*-value based on chi squared test/Fisher exact test; *P*-value < .05 that is considered statistically significant.

In the fully adjusted multivariable logistic regression model (Table 5), several independent predictors remained significant. Participants aged 30 to 39 years (AOR = 4.22, 95% CI: 1.42–12.76) and 40 to 49 years (AOR = 3.64, 95% CI: 1.23–10.89) were significantly more likely to have appropriate HF knowledge compared with those aged < 20 years. Students also showed higher odds of appropriate knowledge (AOR = 3.98, 95% CI: 1.77–9.27), as did participants with higher income (>9000 SR; AOR = 3.43, 95% CI: 1.44–8.44). Notably, working in the medical field remained significant but demonstrated a protective direction (AOR = 0.34, 95% CI: 0.19–0.59). Sex, nationality, education, and HF exposure did not independently predict knowledge in the adjusted model.

**Table 5:** Multiple logistic regression analysis for factors associated with appropriate knowledge of heart failure (n = 399).

Factors		*B*	SE	Wald	*P*-value	AOR	95% CI for AOR	
							Lower	Upper
Age	<20 (ref)							
	20–29	0.737	0.426	2.996	.083	2.09	0.91	4.88
	30–39	1.439	0.559	6.638	.010	4.22	1.42	12.76
	40–49	1.291	0.554	5.425	.020	3.64	1.23	10.89
	50 yr and above	1.176	0.649	3.282	.070	3.24	0.92	11.76
Sex (female)		0.393	0.236	2.775	.096	1.48	0.93	2.36
Nationality (non-Saudi)		1.359	0.822	2.735	.098	3.89	0.92	27.02
Occupation	Not-working (ref)							
	Employee	0.353	0.451	0.611	.434	1.42	0.59	3.47
	Student	1.380	0.421	10.746	.001	3.98	1.77	9.27
Income (SR)	<3000 (ref)							
	3000–6000	0.231	0.463	0.249	.618	1.26	0.51	3.14
	6000–9000	−0.020	0.466	0.002	.965	0.98	0.39	2.45
	>9000 SR	1.233	0.449	7.552	.006	3.43	1.44	8.44
Do you work in the medical field? (yes)		−1.072	0.283	14.369	.000	0.34	0.19	0.59
Education	Below high school (ref)							
	University	0.047	0.557	0.007	.933	1.05	0.36	3.25
	Diploma	−0.537	0.807	0.444	.505	0.58	0.12	2.92
	High school	0.087	0.560	0.024	.876	1.09	0.37	3.41
Do you have HF? (yes)		2.09	1.24	2.84	.092	8.09	0.87	179.98
Do you have HF patient in the family? (yes)		0.08	0.32	0.06	.809	1.08	0.58	2.02

AOR = adjusted odds ratio, *B* = regression coefficient, CI = confidence interval, HF = heart failure, n = number, Ref = reference category, SE = standard error, SR = Saudia Riyals.

* *P*-value based on chi squared test/Fisher exact test; *P*-value < .05 that is considered statistically significant.

## 4. Discussion

The findings of this study revealed both strengths and gaps in HF awareness among adults in Jazan, Saudi Arabia. Key findings include participants’ understanding of HF causes, risk factors, preventive measures, and treatment strategies, as well as associations with sociodemographic factors such as occupation in the medical field and family history of HF. These results provide a foundation for identifying priority areas for targeted educational interventions.

HF is a global health concern affecting individuals of all ages, particularly the elderly, and it causes significant health challenges, reduced quality of life, and even death. In Saudi Arabia, this condition places a substantial burden on the population due to the high costs associated with the treatment and management.^[18-20]^ Individuals employed in the medical field and those with a family member affected by HF demonstrated significantly higher awareness, consistent with previous studies highlighting the influence of medical exposure and personal experience on HF knowledge. However, notable knowledge gaps were identified, particularly regarding the prognosis and severity of the disease. Specifically, less than half (40.4%) of participants were unsure of how HF is caused, while nearly one-third (31.8%) were uncertain about diseases that can lead to HF. Similar knowledge gaps were observed in a Turkish study conducted by Karabulut et al, which found that nearly 3-quarters (71.3%) of participants had inadequate knowledge of HF,^[21]^ suggesting that improved educational initiatives are necessary.

The study highlighted significant concerns regarding awareness of HF symptoms. Only 45.6% of participants identified all 3 major symptoms of HF: shortness of breath, reduced exercise tolerance, and leg edema. This finding aligns with a study by Alshammri et al in Saudi Arabia, which included 385 participants (median age 32 years, 54% female) and was conducted in a region with similar demographic characteristics, where <50% of respondents were aware of HF symptoms.^[15]^ Our findings reported a higher proportion of participants compared with those of a study conducted by Kałużna-Oleksy et al, who observed that only 27% of participants correctly described their understanding of HF symptoms.^[22]^ Individuals without a medical background or prior HF diagnosis may experience delayed presentation and treatment due to misinterpretation of symptoms or underestimation of their severity. These findings are consistent with a study by Zelenak et al in Germany, which also reported poor awareness of HF and the severity of its symptoms.^[16]^

Some previous studies have reported higher levels of HF awareness compared with the present findings. These differences may be attributable to variations in study populations, as several studies focused on patients with established CVD or individuals recruited from healthcare settings, who are more likely to have prior exposure to medical information. In contrast, the present study assessed awareness within the general population. Additionally, differences in assessment tools, scoring thresholds, and regional socioeconomic characteristics may further explain discrepancies in reported knowledge levels.^[13,15-17]^

Our results also indicated that participants with lower income or unemployment had reduced understanding of HF, which aligns with the findings of Jung et al, where a significant proportion of participants with lower income and education demonstrated limited awareness of HF.^[17]^ Furthermore, it was noted that lower income levels were significantly correlated with diminished HF knowledge (*P* = .001), aligning with a Korean study by Kim et al, which indicated that individuals with limited HF awareness had reduced household incomes, resided in rural areas, and possessed lower educational attainment.^[23]^ This underscores the socioeconomic disparities influencing HF awareness within the population.

In terms of sex differences, females (50.5%) were more likely than males (40.7%) to recognize that HF can result from various diseases, including coronary artery disease. This finding aligns with a prior study conducted in Poland by Nowak et al, which reported significantly higher awareness of HF risk factors among females compared to males.^[24]^

The current study revealed that 25% of participants aged 40 to 49 years exhibited appropriate knowledge, while only 8.8% of those aged <20 years and 7.9% of those older than 50 years demonstrated similar knowledge. Most respondents were aged 20 to 29 years (38.6%), likely due to the online survey distribution, which may have contributed to the overrepresentation of younger participants. Multiple studies emphasize the importance of targeting specific demographic groups, such as the elderly, individuals with lower income, and the unemployed, to enhance awareness and improve clinical outcomes related to HF.^[13,25]^

The study noted inadequate awareness about the prevalence of HF, with a considerable proportion (59.6%) of participants uncertain about its commonality, further underscoring the critical need for public health campaigns. Ventura and Piña also emphasized the importance of improving health literacy to enhance outcomes in CVD. These findings suggest that public health efforts to increase HF awareness and health literacy should target both younger populations and vulnerable groups with lower socioeconomic status.^[24-26]^

Overall, this study revealed significant gaps in the general public’s understanding of HF in Jazan, particularly regarding prognosis, symptoms, and risk factors. Our findings are consistent with prior studies in other regions, indicating that HF awareness remains insufficient globally.^[27]^ Targeted education and public health programs could help improve early diagnosis, timely treatment, and clinical outcomes for HF patients in Saudi Arabia. Integrating HF education into routine primary care visits and leveraging media campaigns, similar to the SHAPE campaign in Europe, may further reinforce preventive measures.^[13]^

This study has several limitations. The cross-sectional design limits the ability to infer causal relationships. Data were self-reported and may be subject to recall or social desirability bias. The use of an online survey may have resulted in overrepresentation of younger and more educated individuals, potentially limiting generalizability to older populations. Additionally, the study was conducted in a single geographic region, which may restrict extrapolation of the findings to other regions of Saudi Arabia.

## 5. Conclusion

In conclusion, the study found inadequate knowledge among participants, particularly regarding the most common causes of HF and the measures to reduce the risk of developing it. There was also insufficient knowledge about treatment options, with some participants believing that no treatment was available and that prolonged bed rest was necessary. These findings highlight the need for improved education and awareness programs to address misconceptions and provide accurate information about HF and its management.

## Author contributions

**Conceptualization:** Tariq Al Bahhawi, Alhassan H. Hobani, Mohammed A. Jeraiby, Mostafa Mohrag.

**Data curation:** Ali I. Alharbi, Ali A. Zalah, Faisal H. Almalki, Sawsan H. Alsharif, Ahmed Sayed, Mostafa Mohrag.

**Formal analysis:** Tariq Al Bahhawi, Alhassan H. Hobani, Ahmed Sayed.

**Funding acquisition:** Abdulrahman Hakami.

**Investigation:** Abdulrahman Hakami.

**Methodology:** Tariq Al Bahhawi, Mohamed Salih Mahfouz.

**Project administration:** Luai Alhazmi, Amal H. Mohamed.

**Resources:** Alhassan H. Hobani, Mohammed Somaili, Amal H. Mohamed.

**Software:** Omar Oraibi, Mohammed Somaili, Mohamed Salih Mahfouz.

**Supervision:** Luai Alhazmi, Mohammed Ali Madkhali, Mohammed Somaili.

**Validation:** Omar Oraibi.

**Visualization:** Omar Oraibi, Mohammed Ali Madkhali.

**Writing – review & editing:** Luai Alhazmi, Alhassan H. Hobani.

**Writing – original draft:** Ali I Alharbi, Ali A. Zalah, Faisal H. Almalki, Sawsan H. Alsharif.


