# Supplementary material for: Heart failure awareness and influencing factors among adults in the Jazan, Saudi Arabia
Source: Medicine (Baltimore). 2026 May 15;105(20):e48822. doi: 10.1097/MD.0000000000048822 (PMC13183029; doi:10.1097/MD.0000000000048822)
Supplement: Supplementary file 1 [file medi-105-e48822-s001.docx]

**Questionnaire**

**Demographic characteristics:**

**Age: ...................**

**Gender:**

A- Male

B- Female

**Residency:**

A- Village

B- City

C- Mention…………

**Education level:**

A- Less than high school.

B- High school.

C- College degree.

D- Postgraduate.

**Occupation:** Mention………….

**Medical profession:**

A- yes

B- No

**Exposure to heart failure:**

**Do you have Heart failure?**

A- yes

B- No

**Do you have Heart failure patients in the family?**

A- yes

B- No

**Knowledge of heart failure and symptoms:**

**Which of the following diseases do you know?**

A- AIDS

B- Heart failure

C- coronary heart disease

D- Bronchial carcinoma

E- None

**What is the meaning of HF?**

A- Disturbed blood flow in the heart muscle

B- Defect of the heart valves

C- Failure of the heart

D- Elevated blood pressure

E- I don’t know

**What are typical symptoms or signs of HF?**

A- Shortness of breath under strain

B- Accumulation of water in the legs

C- Weakness of physical performance

D- Pain in right upper arm

E- I don’t know

**Which disease/diseases can lead to HF?**

A- Heart attack

B- Defect of the heart valves

C- Elevated blood pressure

D- Stroke

E- I don’t know

**Which is the most common cause of HF?**

A- Disease of the heart’s coronary blood vessels with disturbed blood flow

B- Lack of sleep

C- Genetic endowments

D- Weight loss

E- I don’t know

**How is HF caused?**

A- By a pumping failure of the heart

B- By the blockage of a brain vessel with a blood clot

C- By the blockage of a venous valve

D- By overweight

E- I don’t know

**Prevalence and age groups:**

**What is the frequency of HF in the population?**

A- There are only single cases

B- It is very rare (less than 0.1%)

C -It is one of the most common diseases in internal medicine

D- Almost everyone will be affected in the course of life

E- I don’t know

**Which age group is especially affected by HF?**

A- Children of up to 12 years

B- Adolescents of up to 18 years

C- Adults of up to 50 years

D- Adults above 65 years

E- I don’t know

**Treatment and prevention:**

**How is the course of HF?**

A- HF mostly goes away by itself

B- It is healed after at least one month of treatment

C- It is about as bad as malignant cancer diseases

D- It can only be healed through surgery

E- I don’t know

**What can I do to reduce my risk of developing HF?**

A- Healthy diet

B- Sufficient physical exercises

C- Not smoke

D- All the above measures

E- I don’t know

**What are the treatment options for HF?**

A- There is no treatment

B- Always strict bed rest for several months

C- Diet

D- Mostly, lifelong treatment with medicines

E- I don’t know

**The awareness state of symptoms of 3 representative cardiovascular diseases:**

**What disease do you think of if someone has chest heaviness that occurs during exertion and disappears with rest?**

A- Angina or MI

B- GI disorders

C- Other heart diseases

D- lung disorders

E- I don’t know

**What disease do you think of if someone has facial paralysis, double vision, and sudden unilateral weakness in the arm?**

A- Stroke

B- Parkinson's disease, epilepsy, other brain diseases

C- Other heart diseases

D- Angina or MI

E- I don’t know

**What disease do you think of if someone has breathlessness, tiredness, and swollen ankles?**

A- Heart failure

B- Heart diseases in general

C- Anginal or MI

D- lung disorders

E- I don’t know
